# Standardized Extract of *Ginkgo biloba* L. Reverses Memory Impairment in Older Female Mice with Basal Forebrain Cholinergic Dysfunction

**DOI:** 10.1007/s11064-026-04829-0

**Published:** 2026-07-14

**Authors:** Beatriz G. Muratori, Carlos R. Caetano, Irina Emanuela T. da Veiga, Andressa G. Soliani, Sofia M. S. e Silva, Juliana P. Gava, Lucinéia F. Ceridório, Luciano Caselli, Rodrigo P. Ureshino, Carla M. Prado, Suzete Maria Cerutti

**Affiliations:** 1 Graduate Program of Structural and Functional Biology, Federal University of São Paulo, São Paulo, SP Brazil; 2 Department of Biological Sciences, Chemical and Pharmaceutical Sciences, Federal University of São Paulo-Institute of Environmental, Campus Diadema, Diadema, SP Brazil; 3 Department of Chemistry, Institute of Environmental, Chemical and Pharmaceutical Sciences, Federal University of São Paulo, Campus Diadema, Diadema, SP Brazil; 4 Department of Biosciences, Federal University of São Paulo, Campus Baixada Santista, Santos, SP Brazil; 5 Graduate Program of Chemical Biology, Federal University of São Paulo, São Paulo, SP Brazil; 6 Cellular and Behavioral Neuropharmacology Laboratory (NeuroCCell), Department of Biological Science, Universidade Federal de São Paulo, São Paulo, SP Brazil

**Keywords:** Aging, Hippocampus, EGb, Acetylcholine, Amyloid-β_1−42_, pTauT231

## Abstract

Aging induces neurochemical changes, particularly in the dentate gyrus (DG), that impair memory. In late-onset Alzheimer’s disease (LOAD), neuronal loss in regions like the entorhinal cortex (EC) and hippocampus proper (HP), along with amyloid-β (Aβ) plaques, tau tangles, and reduced cholinergic signaling, accelerates cognitive decline. Building on our group’s previous findings of cognitive benefits and neuroprotection from *Ginkgo biloba* L. leaf extract (EGb), and considering the limited success of current therapies, EGb has emerged as a promising multi-target strategy for reversing memory impairments. This study evaluates the effects of chronic EGb treatment on memory, anxiety-like behaviors, and motor activity in older female wild-type and VAChT knockdown KD^HET^ and KD^HOM^ mice, which show 45% and 65% reductions in VAChT expression, respectively. We assessed the impact of EGb on Aβ_1−42_ peptide and phosphorylated tau (pTauT231) by quantifying Aβ IR^+^ and pTau IR^+^ cells in the dCA1, dCA3, and dDG, via immunohistochemistry. Results showed that aging led to short- and long-term memory deficits, which were exacerbated in the VAChT KD^HOM^ mice. However, EGb treatment reversed these deficits in a dose-dependent manner by modulating Aβ and pTau-IR^+^ cells in the dCA1, and dDG regions. In vitro, EGb significantly inhibited Aβ aggregation through interactions with Aβ and the POPC monolayer. These findings suggest that EGb may provide a promising therapeutic strategy for AD, improving cognition and offering neuroprotection by targeting key neuropathological features like Aβ plaques and phosphorylated tau.

**Graphical Abstract:**

**Figure:**
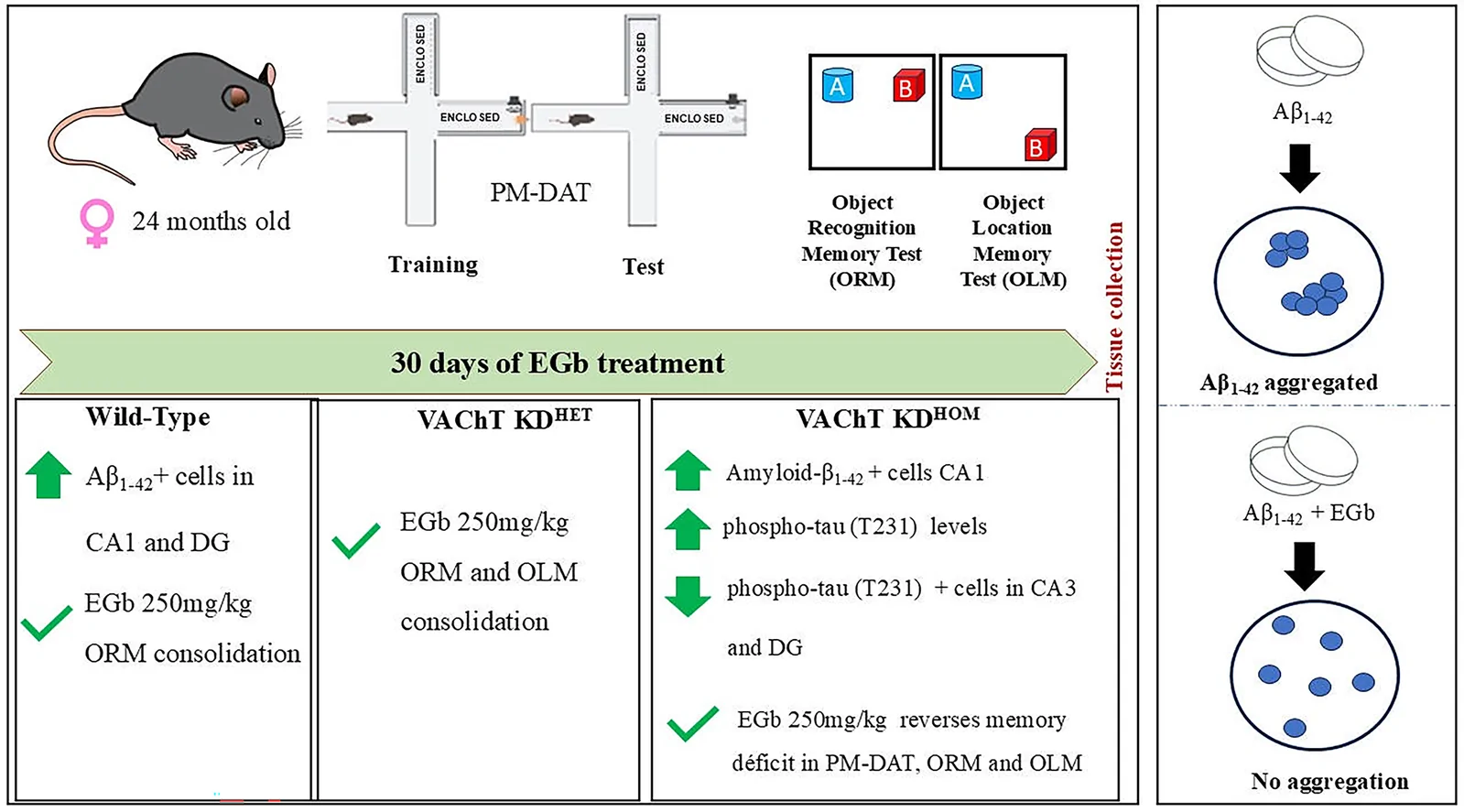

**Supplementary Information:**

The online version contains supplementary material available at 10.1007/s11064-026-04829-0.

## Highlights

- Aging markedly impairs spatial memory in female mice.
- Cholinergic deficits worsen contextual and spatial memory in older mice.
- Low-dose EGb reverses long-term memory deficits in VAChT KD^HOM^ mice.
- EGb decreases pTau231and increases Aβ_1−42_ monomer-IR cells of VACh KD^HOM^ mice.
- EGb reduces the density of Aβ aggregation in vitro.

**Supplementary Information:**

The online version contains supplementary material available at 10.1007/s11064-026-04829-0.

## Introduction

Normative aging is marked by morphological, cellular, and molecular changes that primarily affect the functionality of key regions in the central nervous system. These alterations disrupt the physiological processes of neuronal and glial cells, leading to a gradual decline in memory and cognitive function, typically becoming noticeable after the age of 65–70 [1–4]. Both human and nonhuman behavioral studies have demonstrated age-related declines in performance on tasks involving hippocampal formation (HF), supporting the hypothesis that hippocampal plasticity and memory are impaired during normal aging [3, 5]. Structural and functional changes in the HF include progressive loss of dendritic surface in neurons of the dentate gyrus (DG) granule cells, altered cellular connectivity, dysregulation of calcium (Ca^2+)^ levels, and changes in gene expression, all of which impact the dynamics of hippocampal neuronal ensembles. In addition, CA3 pyramidal cells exhibit reduced synaptophysin staining in their dendritic regions and significant neuronal excitability, similar to observations in the DG [6, 7]. Further studies indicate that hippocampal neurons in older rodents have a diminished ability to sustain long-term potentiation (LTP), a critical mechanism for long-term memory (LTM) [6, 8]. Elderly animals are valuable models for distinguishing between normal and pathological aging [7, 9–12]. Thus, cognitive decline in aging is primarily linked with the loss of dendritic spines and synaptic efficiency, rather than a reduction in neuronal number. Nevertheless, the exact mechanisms driving these changes remain largely elusive [6, 13].

Complementary data from our group have sought to explain age-related alterations. We demonstrated that new information (common novelty) can be linked to, or incorporated into, a short-lasting fear memory through a brief temporal window, thereby inducing memory consolidation. Importantly, aging alters patterns of neuronal activity and overlap, resulting in impaired novelty-induced consolidation [14]. In contrast, pathological aging, as seen in late-onset Alzheimer’s disease (LOAD), involves distinct molecular and functional alterations. For example, Neuner et al. (2017) showed that 5XFAD mice, a widely used AD model, exhibit impaired trace fear memory and altered neuronal membrane properties compared to normally aging mice. Importantly, they identified proteins specifically associated with normal aging that differ from those linked to AD. One such protein is the repressor element 1-silencing transcription factor (REST), which participates in chromatin remodeling and represses genes involved in synaptic function. These findings highlight critical differences between normative aging and the progression of LOAD [15].

LOAD is a neurodegenerative progressive condition, with aging being the primary risk factor, especially prevalent in women. Its hallmark features include the accumulation of misfolded amyloid-β (Aβ) plaques, and the formation of neurofibrillary tangles (NFTs) composed of hyperphosphorylated tau [16, 17]. Alterations in glial cell function have been identified as playing a central role in the advancement of AD [18]. AD prominently affects the EC, CA1, and the subiculum [19, 20], impacting behavior through complex interactions with other brain areas. The prefrontal cortex, essential for memory and executive function, undergoes significant age-related decline, marked by notable cell loss [2]. This region also receives direct cholinergic projections from the basal forebrain, the primary source of acetylcholine (ACh) production [21]. Disruption of cholinergic signaling in AD contributes to deficits in attention, decision-making, and memory formation. VAChT (Vesicular Acetylcholine Transporter) knockdown mice are widely used to investigate cholinergic deficits in neurodegenerative conditions such as AD. These models allow detailed analysis of synaptic alterations, memory deficits, and behavioral changes, although they do not naturally develop Aβ plaques or NFTs [22, 23]. Studies indicate that Aβ peptides and neurofibrillary tangles (NFTs) alter nicotinic and muscarinic receptors, disrupting cholinergic signaling [24, 25]. Reduced cholinergic activity in hippocampal neurons has also been associated with increased expression of gap junction proteins, potentially altering muscarinic receptor conformation [10]. In vitro studies have highlighted the interaction between β-amyloid and lipid membranes, particularly 1-palmitoyl-2-oleoyl-sn-glycero-3-phosphocholine (POPC), a key component of neuronal membranes [26, 27], which has been extensively studied for its interactions with Aβ peptides [28–31]. Notably, POPC has shown significant binding with Aβ, suggesting that membrane structural alterations contribute to AD progression [28–31].

Previous reports showing estrogen receptor α (ERα) immunoreactivity in VAChT-labeled terminals suggest that estrogens can influence the responsiveness of septohippocampal cholinergic afferents to trophic factors [32]. Estrogen has also been shown to increase TrkA receptor expression in cholinergic septal neurons (Gibbs and Aggarwal, 1998) while NGF binding can potentiate acetylcholine release [33]. Furthermore, TrkA immunoreactivity, like VAChT and ERα, is primarily associated with clusters of small synaptic vesicles in presynaptic cholinergic profiles [34], supporting potential local interactions between estrogen signaling and cholinergic function. Moreover, a decline in estrogen levels in older women reduces neuroprotection, thereby increasing brain vulnerability to disease [35]. Because estrogen modulates cholinergic signaling, which declines with age, VAChT KD mice are a suitable model to investigate how cholinergic hypofunction interacts with estrogenic signaling to impair memory. This model also enabled us to evaluate EGb, a multitarget extract, for its potential to modulate these pathways.

Currently, several treatments are under investigation to alleviate cognitive impairment in AD, including Donepezil, which targets the cholinergic system by inhibiting acetylcholinesterase and preserving ACh levels in the synaptic cleft. However, due to the complex nature of AD, existing medications are insufficient, highlighting the need for new therapies, potentially through a multi-target approach. Studies have demonstrated that flavonoid-rich plants, including the standardized extract of *Ginkgo biloba* L. leaves (EGb), may offer protective benefits against cognitive decline and memory loss associated with aging and AD. Studies from our group have shown that EGb reverses conditioned suppression memory impairment in a dose-dependent manner after blocking 5HT_1A_ or GluN2B-NMDA receptors [36]. Moreover, EGb enhances short-term discriminative avoidance memory and exhibits preventive effects on the prefrontal cortex in middle-aged rats [37]. EGb also modulates protein expression in the dHF, affecting processes such as cytoskeleton remodeling, dendritic spine stability, myelin sheath formation, and tight junction composition crucial for long-term memory (LTM). It also exerts neuroprotective and anti-apoptotic effects [38]. Additionally, EGb regulates BDNF expression in the hippocampus, supporting the persistence of recognition memory [39] and enhancing the consolidation process and maintenance of memory [40, 41]. Object recognition memory (ORM), object location memory (OLM), and the plus-maze discriminative avoidance task (PMDAT) are established models used to study recognition memory, spatial memory, and conditioned learning, respectively. These tasks are widely employed to investigate age-related cognitive decline, anxiety-like behaviors, and spontaneous locomotor activity, regardless of whether they are associated with degenerative processes, as they depend on the functional integrity of structures within the HF. In a previous study, our group found that EGb improved cognitive function by modulating VAChT levels and increasing the number of VAChT-positive pyramidal neurons in the hippocampus of young female mice [42]. Furthermore, EGb was able to reverse cognitive impairments in young VAChT KD^HOM^ female mice. This study aims to further investigate the effects of EGb on memory in older female mice with varying levels of cholinergic dysfunction due to VAChT reduction. We also examined how EGb influences amyloid-β accumulation and tau phosphorylation, two critical factors in AD pathophysiology, using protein expression analysis and in vitro studies.

## Materials and Methods

### Animals

VAChT mutant mice were generated following the method outlined by Prado et al. [43]. Female mice 20–30 g aged 24 months, knockdown homozygous for VAChT (VAChT KD^HOM^) exhibited a 65–70% decrease in VAChT gene expression, while heterozygous (VAChT KD^HET^) mice showed a 45% decrease. Both mutant and C57BL/6 wild-type (WT) mice, with similar genetic backgrounds, were acquired from the Animal Facility at the University of São Paulo School of Medicine. They were housed in the animal facility of the Federal University of São Paulo, maintained at 21–23 °C with a 12-h light/dark cycle. They had ad libitum access to water and food. The experiments were conducted during the light phase of the cycle. The experimental procedures were obtained from the local Committee for Ethics in Animal Experimentation at the Federal University of São Paulo (CEUA, approval no. 7387221217) and NIH guidelines for the Care and Use of Nonhuman Animals in Research (NIH publication #85-23, revised in 1985).

### Experimental Procedure and Drugs Administration

Each genotype (WT littermate controls and VAChT knockdown [KD^HET^ or KD^HOM^]) was further distributed into six groups (*n* = 6–8 per group) according to the following treatments: (1) naïve (no treatment and no behavioral procedures); (2) 0.9% saline (vehicle); (3) 5 mg/kg Donepezil (positive control); and (4–6) EGb at doses of 250 mg/kg, 500 mg/kg, and 1000 mg/kg. This resulted in a total of 18 groups. The standardized extract of *Ginkgo biloba* L. (EGb), obtained from green leaves and containing 24% flavonoid glycosides, 6% terpene lactones, and < 5 ppm ginkgolic acid (from Huacheng Biotech Inc., China, lot GK-100708), was used in all experiments. The bioactive compounds in the extract were previously identified by our group using UHPLC-HRMS/MS and molecular networking [41]. Moreover, previous studies from our laboratory [38, 41, 42] together with reports from the literature [44, 45], have demonstrated that the biological effects of EGb are dose- and treatment-dependent. Specifically, doses that are effective after acute administration may differ from those required following chronic treatment. Therefore, the doses used in the present study were selected based on previous findings from our laboratory [36, 38, 42, 46–48]. EGb was diluted in 0.9% saline and administered orally at a volume adjusted to each animal’s body weight, not exceeding 3% of body weight, which corresponds to the maximum gastric volume tolerated by mice. All mice’s body weight was observed during the 30 days of treatment, and no changes were observed.

The estrous cycle was monitored in all mice during both training and testing sessions across the memory evaluation paradigms. During preliminary PMDAT sessions (an aversive memory task), we observed that the estrous cycle phase significantly influenced performance, particularly compared to ORM and OLM tasks. Control mice performed better during proestrus and worse during the estrus–diestrus phases. Based on these observations, we routinely assessed the cycle phase before each behavioral session. If an animal was in a phase likely to affect performance, the testing session was postponed to the following day.

### Behavioral Analysis

#### Plus-Maze Discriminative Avoidance Task (PMDAT)

The apparatus was constructed from wood and comprised two enclosed arms (EA) opposite two open arms (OA), arranged in an L-shape. One enclosed arm, referred to as the aversive enclosed arm (AEA), was used to deliver aversive stimuli, including a 100-watt lamp and 85 dB noise, while the other enclosed arm (NAEA) served as the non-aversive control. On Day 23, following treatment, the mice were acclimated in a low-light room for 5 min before the training session, where each mouse was placed in the center of the apparatus for 10 min. Aversive stimuli were triggered when the mouse entered the AEA with all four paws. A test session took place 24 h later, lasting 3 min, without the application of aversive stimuli. During the first minute of the test session, the latency for mice to enter the AEA was recorded. Memory retention was measured by the percentage of time spent in the AEA, calculated as: % time in AEA = [(time in AEA)/(time in both enclosed arms)] × 100. Anxiety-like behavior was assessed using the anxiety index (AI), which considered the total time spent and total entries into the open arms per mouse calculated as: AI = 100 − [(OAs time/total time) + (OA entries/total entries)/2] during the training session [49]. while spontaneous locomotor activity was measured by tracking the total number of NAEA entries throughout the training session. Behavioral sessions were recorded and analyzed using the Insight^®^ Aversive Maze Program. The apparatus was cleaned with a 20% ethanol solution between animals and sessions.

#### Object Recognition and Object Location Memory Tasks

From days 26 to 30, following the initiation of drug treatment, female mice underwent tests for object location memory (OLM) and object recognition memory (ORM) to assess non-aversive, hippocampus-dependent memory. On day 26, each mouse was habituated for 15 min in an empty arena. On days 27 and 28, two separate 15-min training sessions were conducted, during which two identical “sample objects” (A and A′) were placed on opposite sides of the arena, 10 cm from the walls. A time cut of 20 s of exploration in both objects during training was considered to include animals’data. On day 29, during the ORM test, one sample object (A′) was replaced with a novel object (B), and each mouse explored both objects for 10 min. On day 30, during the OLM test, objects A and B were reintroduced, with object B positioned in a new diagonal position. After each session, the arena and objects were cleaned with 20% ethanol. The Discrimination Index (DI) was calculated as follows: DI = [(B − A)/(A + B)], with DI values ranging from − 1 to 1 [40, 50]. Exploration was defined as the time spent sniffing or touching the object with the nose and/or forepaws [51–53]. The Discrimination Index (DI) was calculated as: DI = [(B − A)/(B + A)], where B is the time spent exploring the novel or the moved object and A is the time spent exploring the familiar or stationary object. DI values range from − 1 to 1, with DI = 0 indicating equal exploration of both objects. Values above 0 indicate preference for the novel or moved object, while values below 0 indicate preference for the familiar or stationary object. Total object exploration time and the frequency of object contacts were analyzed during both the training and test sessions. No significant differences in total object exploration time were observed among the experimental groups during the training session. In addition, a minimum exploration criterion was applied, and only animals that accumulated at least 20 s of exploration across both objects (A + A′) during the training session were included in the analyses. Total object exploration time during the training session is now presented in the Supplementary Information (Supplementary Fig. 1). For more details of behavioral tests, see our previous work by Muratori and colleagues [42].

### Immunohistochemical Analysis

The brains were then postfixed in the same solution and cryoprotected in 10% sucrose-0.1 M PBS (pH 6.8) for 72 h before being stored at − 80 °C. Coronal section  (16 μm thick) were cut using a cryostat (Leica, CM1850 UV, Germany) and mounted on gel-coated slides for analysis (*n* = 4/group).

The sections were processed through a series of immunohistochemical steps: rehydration, washing in PBS (pH 7.6), and blocking with 3% hydrogen peroxide in water. Non-specific binding was prevented using 1% BSA in 0.1 M PBS (pH 7.6). Following incubation with the primary antibody as follows: anti-monomers of amyloid-*β*_1−42_ (D9A3A; dilution 1:1500) from Cell Signaling Technology^®^ and anti-phospho tauT231 (ab151559; dilution 1:300) from Abcam^®^, the sections were washed and followed by incubation with biotinylated goat anti-rabbit antibodies. Immunodetection was performed using the Labeled StreptAvidin HRP system (Diagnostic BioSystems, Pleasanton, USA) and DAB substrate (DAB Polymer RE 7270-CE, Leica Biosystems, Germany). *C*oronal sections corresponding to the dorsal hippocampus along the rostrocaudal axis (AP -1,43 mm to -2,69 mm from bregma, according to Paxinos and Franklin mouse brain atlas [54] were analyzed. The dorsal CA1 (dCA1), dorsal CA3 (dCA3), and dentate gyrus (DG) were identified based on the anatomical boundaries defined in the corresponding atlas plates. The sections were then mounted for permanent preservation. Amyloid-β_1−42_ and pTau231-positive cells were counted bilaterally in the pyramidal layers of CA1 and CA3, and in the granular layer of the dentate gyrus (DG). Three sections from each region were analyzed, and three subfields within each region were selected, covering both rostral and caudal levels based on the Franklin and Paxinos atlas [54]. Immunoreactive (IR+) cells were manually counted within a predefined area of 94,566 μm² in each hippocampal subregion using NIH ImageJ software. Images were obtained using a Zeiss Imager A2 microscope using a Plan Neofluar 40x/0.75 objective and analyzed with Zen software.

### Analysis of EGb Effects on Amyloid-β: In Vitro Study

A Langmuir trough (KSV Instruments), equipped with a hydrophobic rectangular compartment, movable barriers, and a liquid surface pressure sensor, enabled control over the molecular area and surface pressure of the monolayers to simulate biological membranes [31]. This apparatus also facilitated various methods for characterizing the films. POPC phospholipid was sourced from Avanti Polar Lipids and dissolved in chloroform (Synth) to achieve a concentration of 0.16 mg/mL. The same EGb used in behavioral analyses was dissolved in purified water using a Milli-Q System to a concentration of 0.125 mg/mL [55]. Aβ protein fragment 1–42 (Aβ_1−42_) was obtained from Sigma-Aldrich and dissolved in a phosphate buffer solution (PBS) to a concentration of 0.5 mg/mL. A PBS solution (0.5 M, pH = 7.6) served as the aqueous subphase in experiments to mimic the extracellular cellular environment.

The POPC monolayer was prepared by spreading specific aliquots of the organic solution onto the buffer-air interface in a Langmuir trough (micro trough from KSV Instruments, total volume 40 mL). After allowing 15 min for solvent evaporation, 100, 250, 500, or 1000 µL of EGb was injected into the subphase a few millimeters below the monolayer and allowed to stabilize. Compression of the film was performed using two asymmetric barriers at a rate of 10 mm/min. The procedure was repeated with the addition of 10 µL of Aβ to the subphase. During compression, the surface pressure (π) was measured by decreasing the monolayer area (A), using a paper filter as a Wilhelmy plate, to obtain π-A isotherms (25 ± 1 °C). Brewster Angle Microscopy (BAM) images were captured using a mini-BAM (KSV Instruments) with the monolayers compressed to stability. Langmuir films provide a valuable method for investigating EGb in the context of AD, as they mimic biological membranes with structural and organizational similarities [56]. These films involve spreading amphiphilic compounds on an aqueous subphase, forming a nanostructured monolayer at the air-water interface. Amyloid peptides are reported to interact with specific lipids [57].

### Statistical Analysis

The normality of the data was initially evaluated using the Kolmogorov–Smirnov test. Parametric tests were then applied to evaluate the total time spent and the number of entries into the enclosed arms (AEA vs. NAEA) and the open arms (OA) to assess discriminative avoidance memory and the anxiety index. Between-group and genotype comparisons for both behavioral and protein analyses were conducted using one-way ANOVA followed by Tukey’s post-hoc test. e *t*-test was applied to assess whether the discrimination index (DI) was significantly greater than chance (DI > 0). GraphPad Prism 10 was employed for statistical analysis (GraphPad Software, San Diego, CA, USA), with a significance set at α = 0.05. Data are reported as mean ± S.E.M. The size effect for mean differences was assessed using Cohen’s d, and it was determined by d = (M1 – M2)/s pooled; where M1 = mean of group 1; M2 = mean of group 2 and s pooled standard deviations for the two groups. The values are interpreted according to thresholds of 0.20 (small), 0.50 (medium), and 0.80 (large).

## Results

### Impact of Normal Aging on Hippocampal-Dependent Memory

**Fig. 1:**
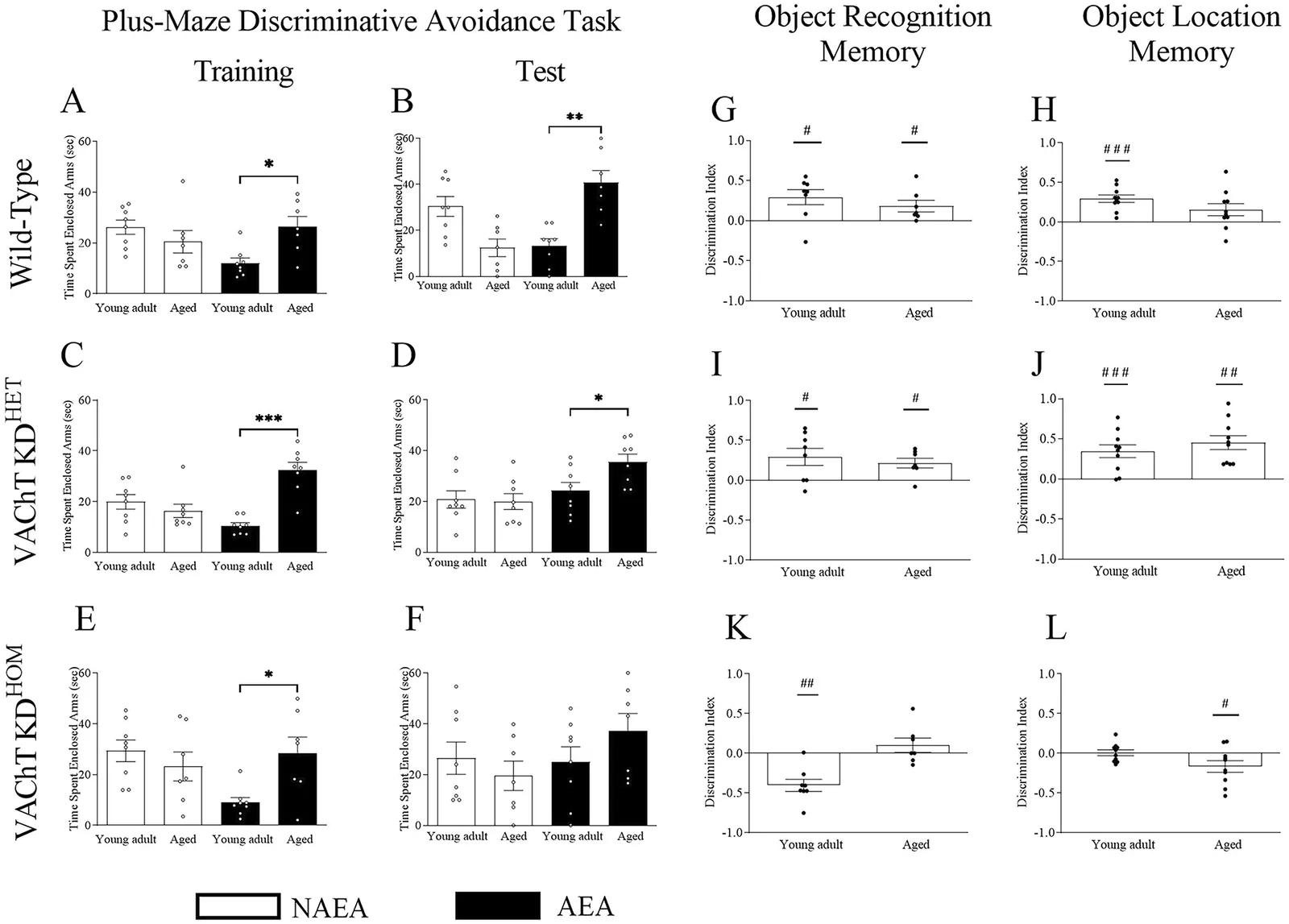
Comparative data between young adult and older females of three genotypes Wild Type, VAChT KD^HET^, and KD^HOM^ submitted to Plus-Maze Discriminative Avoidance Task (PMDAT) (**A–F**) and to Object Recognition and Location Tasks (**G–L**). PMDAT was analyzed using the comparative time spent in enclosed arms (non-aversive—white bars vs. aversive—black bars). ORM and OLM were assessed using the Discrimination Index (DI). Comparisons between groups are denoted as follows: *P < 0.05, **P < 0.01, ***P < 0.001, according to the Paired T-Test. The horizontal lines represent the chance value for DI (0.0). # Represent P values to the ratio discriminations from the group above chance level, according to One-Sample *t*-test #*P* < 0; ##*P* < 0.01 or ###*P* < 0.001 (*n* = 6–8/group)

**Fig. 2:**
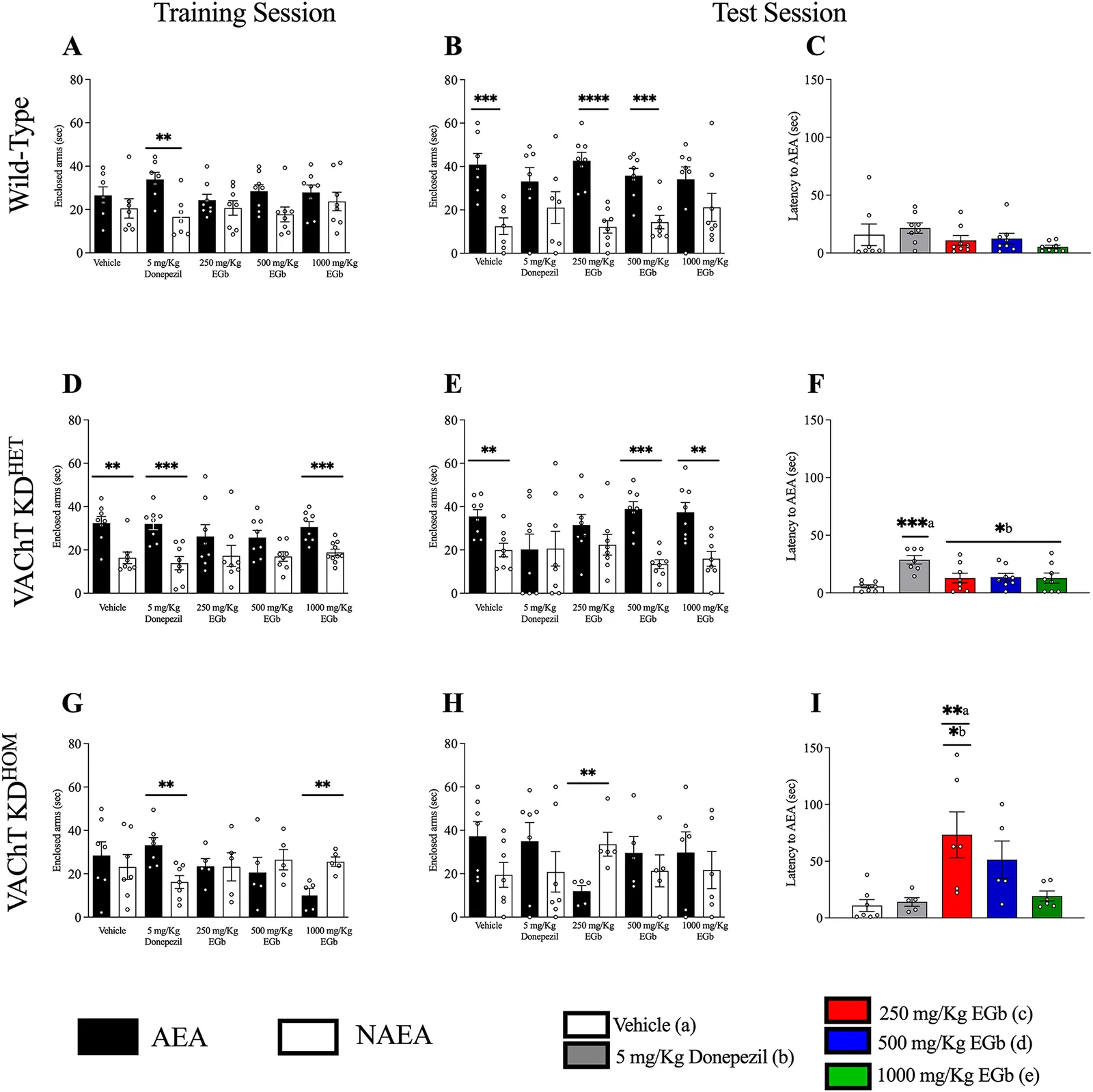
The time spent in enclosed arms [non-aversive enclosed arm (NAEA, white bars) and aversive enclosed arm (AEA, black bars)] during training (short-term memory) and test sessions (long-term memory) in adult older female mice WT (**A**, **B**) VAChT KD^HET^ (**D**, **E**) and KD^HOM^ (**G**, **H**) during the Plus-Maze Discriminative Avoidance Task (PMDAT). Treatments included vehicle, EGb (250, 500, or 100 mg/kg), or Donepezil (5 mg/kg) administered via gavage for 1–24 days before the experimental sessions. The latency to enter the AEA in the test session was assessed in WT (**C**), VAChT KD^HET^ (**F**), and VAChT KD^HOM^ (**I**) mice. Comparisons between groups are denoted as follows: *P < 0.05, **P < 0.01, ***P < 0.001, and ****P < 0.0001, according to Paired *t*-Test and One-way ANOVA with post hoc Tukey’s multiple comparison test (*n* = 6–8/group)

To further investigate the effects of aging on STM and LTM discriminative avoidance memory, as well as recognition memory (ORM and OLM), we revisited data from our lab using young female mice of the same model [42]. Comparisons between the previous dataset and the current study were performed using paired *t*-tests for both training (Fig. 1A, C, E) and testing (Fig. 1B, D, F) sessions in vehicle-treated control animals. Our data revealed that older female mice treated with vehicle spent significantly more time in the AEA than young adult mice. This effect was observed in during the training sessions [WT *t*(6) = 2.80; (*P* = 0.030); Cohen’s d = 2.32; VAChT KD^HET^ (*t*(6) = 5.470; *P* = 0.0009; Cohen’s d = 5.89); VAChT KD^HOM^ (*t*(7) = 2.576; *P* = 0.042); Cohen’s d = 3.55] and during the testing session [WT (*t*(6) = 4.016; *P* = 0.007; Cohen’s d = 2.92); VAChT KD^HET^ (*t*(6) = 2.725; *P* = 0.029; Cohen’s d = 2.27)], except for the VAChT KD^HOM^ group, in which no significant age-related difference was observed during the test session (*t*(6) = 1.023; *P* = 0.345; Cohen’s d = 1.66). These findings suggest that both young and older VAChT KD^HOM^ mice show impairments in LTM for discriminative avoidance memory. Further analysis revealed that older mice spent similar amounts of time within both AEA and NAEA during the training session [WT (*t*(6) = 0.723; *P* = 0.491); VAChT KD^HOM^ (*t*(6) = 0.6124; *P* = 0.5517)]. Likewise, aged VAChT KD^HOM^ mice also spent similar amounts of time in both enclosed arms during the test session [*t*(6) = 1.991, *P* = 0.018]. Moreover, older WT mice spent significantly more time in AEA during the testing session (*t*(6) = 3.202; *P* = 0.0009), whereas aged VAChT KD^HET^ mice spent significantly more time in the AEA during both the training (*t*(6) = 3.961; *P* = 0.0014) and test (*t*(6) = 3.517; *P* = 0.034) sessions. Together, these findings indicate that aging impairs both short-term and long-term discriminative avoidance memory (Fig. 2).

Similarly, analysis of non-aversive memory showed no significant differences in Discrimination Index (DI) between young and older female mice in the WT genotype during ORM (*t*(6) = 1.258; *P* = 0.2550) and OLM (*t*(6) = 1.807; *P* = 0,2609) testing sessions (Fig. 1G, H). Further analysis using a one-sample *t*-test revealed that DI values significantly exceeded chance levels (DI > 0), indicating that both young ( *t*(7) = 3.150; *P* = 0.0161; DI = 0.2947) and older mice (*t*(6) = 2.510; *P* = 0.0459) in the WT group successfully learned ORM, with no aging-related decline in performance. Moreover, young mice (*t*(7) = 1.994; *P* = 0.0001; DI = 0.2928), but not older mice (*t*(6) = 1.640; *P* = 0.1522; DI = 0.1541), were able to discriminate the moved object during the OLM testing session. Data from the VAChT KD^HET^ genotype revealed no significant differences between young and older female mice during ORM (*t*(6) = 1.232; *P* = 0.2641) and OLM (*t*(6) = 1.1581; *P* = 0.2910) testing sessions (Fig. 1I, J). Additionally, DI values significantly exceeded chance levels (DI > 0), indicating that both young (*t*(7) = 2.738; *P* = 0.029; DI = 0.2935) and older mice (*t*(7) = 3.462; *P* = 0.013; DI = 0.2144) successfully learned ORM as well as OLM [young (*t*(7) = 3.660; *P* = 0.008; DI = 0.3478), older (*t*(6) = 8.329; *P* = 0.0002; DI = 0.4541)].

However, comparisons between young and aged VAChT KD^HOM^ mice revealed a significant difference in ORM performance (*t*(6) = 3.576; *P* = 0.0011) but not in OLM performance (*t*(6) = 0.3044; *P* = 0.7711). One-sample *t*-tests further showed that both young (*t*(7) = 5.300; *P* = 0.011, DI = − 0.4041) and older mice (*t*(6) = 1.125; *P* = 0.1023) failed to discriminate between the familiar and novel objects during the ORM test, with discrimination index (DI) values that were below or not different from chance level (DI ≤ 0). These findings indicate that the more pronounced hypocholinergic state of the VAChT KD^HOM^ genotype impaired object recognition memory regardless of age. A similar pattern was observed during the OLM test. Young mice (*t*(7) = 0.02956; *P* = 0.9772; DI = 0.005) failed to discriminate the relocated object, with discrimination index (DI) values not significantly different from chance level. Likewise, older mice (*t*(6) = 2.283; *P* = 0.04; DI = − 0.1675) also failed to discriminate the relocated object, exhibiting DI values below chance level. Together, these findings indicate impaired object location memory in both young and aged VAChT KD^HOM^ mice.

### EGb Enhances Hippocampal-Dependent Memory in Older Mice in a Dose-Dependent Manner

#### Aversive Memory

Given the age-related deficits in discriminative avoidance memory and previous findings from our laboratory demonstrating the beneficial effects of EGb on memory, we investigated whether EGb treatment administered before memory acquisition could attenuate these deficits in older WT mice (Fig. 2A, B). Within-group comparisons using paired *t*-tests revealed that none of the treatments were able to reverse the deficits in aversive memory. Mice treated with EGb at 250 mg/kg (*t*(7) = 0.6090; *P* = 0.5617); 500 mg/kg (*t*(7) = 1.762; *P* = 0.1214) and 1000 mg/kg (*t*(7) = 0.5968; *P* = 0.5956) failed to discriminate between the aversive enclosed arm (AEA) and the non-aversive enclosed arm (NAEA) during the training session. Notably, mice treated with 5 mg/kg Donepezil (*t*(6) = 2.840; *P* = 0.0296) aspent significantly more time in the AEA during the training session. During the test session, vehicle-treated mice exhibited impaired memory, spending significantly more time in the AEA (*t*(6) = 3.202; *P* = 0.0185). Similarly, EGb failed to reverse the age-related memory deficits, as mice treated with EGb at 250 mg/kg (*t*(7) = 4.562; *P* < 0.0026); and 500 mg/kg (*t*(7) = 4.639; *P* = 0.0114)] also spent significantly more time in the AEA during the test session. Moreover, mice treated with EGb at 1000 mg/kg failed to discriminate between the AEA and the NAEA (*t*(7) = 1.037; *P* = 0.323).

In older VAChT KD^HET^ mice (Fig. 2D, E), neither Donepezil nor EGb treatment restored discriminative avoidance memory. During the training session, vehicle-treated mice (*t*(7) = 3.024; *P* = 0.019), Donepezil-treated mice (*t*(7) = 3.719; *P* = 0.0075), and mice treated with EGb at 1000 mg/kg (*t*(7) = 3.441; *P* = 0.018) spent significantly more time in the aversive enclosed arm (AEA). Likewise, mice treated with EGb at 250 mg/kg (*t*(7) = 0.3710; *P* = 0.9558) or 500 mg/kg (*t*(7) = 1.658; *P* = 0.1413) failed to discriminate between the aversive (AEA) and non-aversive enclosed arms (NAEA). Together, these findings indicate that none of the treatments restored short-term discriminative avoidance memory in aged VAChT KD^HET^ mice. Consistently, during the test session, vehicle-treated mice (*t*(7) = 2.540; *P* = 0.038) continued to spend significantly more time in the AEA, indicating impaired long-term discriminative avoidance memory. A similar behavioral profile was observed in mice treated with EGb at 500 mg/kg (*t*(7) = 4.698; *P* = 0.0022) and 1000 mg/kg (*t*(7) = 2.756; *P* = 0.0282), which also spent significantly more time in the AEA. Likewise, mice treated with Donepezil (*t*(7) = 0.0345; *P* = 0.9734), and EGb (250 mg/kg (*t*(7) = 0.6792; *P* = 0.3657) spent similar time in both enclosed arms. Similarly, mice treated with Donepezil (*t*(7) = 0.0345; *P* = 0.9734) or EGb at 250 mg/kg (*t*(7) = 0.6792; *P* = 0.3657) spent similar amounts of time in the AEA and the non-aversive enclosed arm (NAEA), indicating that they failed to discriminate between the two enclosed arms. Together, these findings demonstrate that none of the treatments restored short- and long-term discriminative avoidance memory in aged VAChT KD^HET^ mice.

In aged VAChT KD^HET^ mice (Fig. 2D, E), neither Donepezil nor EGb treatment restored discriminative avoidance memory. During the training session, vehicle-treated mice (*t*(7) = 3.024; *P* = 0.019), Donepezil-treated mice (*t*(7) = 3.719; *P* = 0.0075), and mice treated with EGb at 1000 mg/kg (*t*(7) = 3.441; *P* = 0.018) spent significantly more time in the aversive enclosed arm (AEA). In addition, mice treated with EGb at 250 mg/kg (*t*(7) = 0.3710; *P* = 0.9558) or 500 mg/kg (*t*(7) = 1.658; *P* = 0.1413) failed to discriminate between the aversive (AEA) and non-aversive enclosed arms (NAEA). Together, these findings indicate that none of the treatments restored short-term discriminative avoidance memory in aged VAChT KD^HET^ mice. During the test session, vehicle-treated mice (*t*(7) = 2.540; *P* = 0.038) continued to spend significantly more time in the AEA, indicating impaired long-term discriminative avoidance memory. A comparable behavioral profile was observed in mice treated with EGb at 500 mg/kg (*t*(7) = 4.698; *P* = 0.0022) and 1000 mg/kg (*t*(7) = 2.756; *P* = 0.0282), which also spent significantly more time in the AEA. In contrast, mice treated with Donepezil (*t*(7) = 0.0345; *P* = 0.9734), and EGb (250 mg/kg (*t*(7) = 0.6792; *P* = 0.3657) spent similar amounts of time in the AEA and NAEA, indicating that they failed to discriminate between the two enclosed arms. Together, these findings demonstrate that none of the treatments restored long-term discriminative avoidance memory in aged VAChT KD^HET^ mice.

A distinct behavioral profile was observed in aged VAChT KD^HOM^ mice (Fig. 2G, H), revealing dose-dependent effects of EGb on discriminative avoidance memory. During the training session, vehicle-treated mice (*t*(6) = 0.4416, *P* = 0.6743) and mice treated with EGb at 250 mg/kg (*t*(4) = 0.9177, *P* = 0.4264) or 500 mg/kg (*t*(4) = 0.5132, *P* = 0.6349) spent similar amounts of time in the AEA and the NAEA, indicating impaired short-term memory. Likewise, Donepezil-treated mice also failed to improve memory acquisition, spending significantly more time in the AEA (*t*(6) = 2.884, *P* = 0.028). In contrast, mice treated with EGb at 1000 mg/kg spent significantly more time in the NAEA (*t*(4) = 4.169, *P* = 0.0031, Cohen’s *d* = 1.61), indicating successful acquisition of discriminative avoidance memory. During the test session, vehicle-treated mice (*t*(6) = 1.422, *P* = 0.2048), Donepezil-treated mice (*t*(6) = 0.7833, *P* = 0.4632), and mice treated with EGb at 500 mg/kg (*t*(4) = 0.5902, *P* = 0.5868) or 1000 mg/kg (*t*(4) = 0.4474, *P* = 0.6733) spent similar amounts of time in the AEA and NAEA, indicating impaired long-term memory. In contrast, mice treated with EGb at 250 mg/kg spent significantly more time in the NAEA (*t*(4) = 2.873, *P* = 0.0453, Cohen’s *d* = 1.73), indicating successful long-term memory retention. Together, these findings demonstrate dose-dependent effects of EGb in aged VAChT KDHOM mice, with the highest dose (1000 mg/kg) improving short-term memory acquisition, whereas the lowest dose (250 mg/kg) restored long-term memory retention.

To further investigate the effects of aging and reduced cholinergic tone on aversive memory, we assessed the latency to the animals’ first entry into the AEA during the test session. Analysis using one-way ANOVA revealed no significant differences in the latency to the first entry into the AEA among treatment groups in WT mice (F_4, 34_ = 1.364; *P* = 0.2668) (Fig. 2C). In contrast, older VAChT KD^HET^ mice (Fig. 2F) treated with Donepezil showed a significantly longer latency to enter the AEA than vehicle-treated mice and those treated with EGb at 250, 500, or 1000 mg/kg (F_4, 35_ = 5.205; *P* = 0.0021). In older VAChT KD^HOM^ mice (Fig. 2I), treatment with EGb at 250 mg/kg significantly increased the latency to enter the AEA compared with both the vehicle and Donepezil groups (F_4, 35_ =5.205; *P* = 0.002; Cohen’s d = 1.87), indicating improved long-term discriminative avoidance memory.

#### Non-aversive Memory

**Fig. 3:**
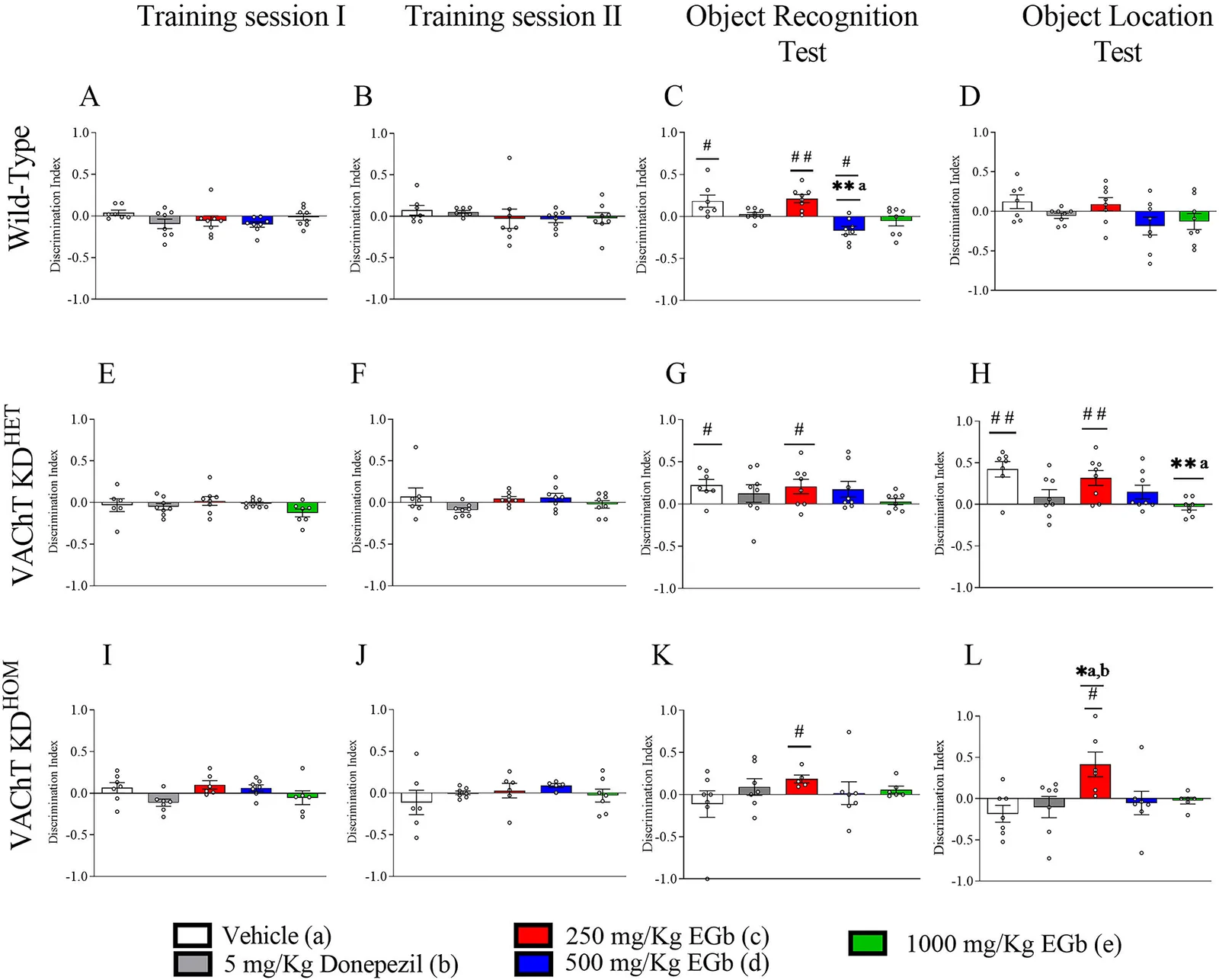
The Discrimination Index (DI) for sample objects was evaluated during training sessions, as well as for both novel and sample objects in the Object Recognition Memory test (ORM), and for novel and sample locations in the Object Location Memory test (OLM) across WT, VAChT KD^HET^, and VAChT KD^HOM^ mouse genotypes for WT, VAChT KD^HET,^ and VAChT KD^HOM^ mice genotypes of control groups [vehicle (0.9% saline) or 5 mg/Kg Donepezil)] and treated with EGb at doses 250 mg/Kg, 500 mg/Kg and 1000 mg/Kg. Comparisons between groups are denoted as follows: *P < 0.05, **P < 0.01 according to One-way ANOVA with post hoc Tukey’s multiple comparison test. ^#^*P* < 0 or ^##^*P* < 0.01 means comparisons of each group in chance level “0.0” according to One-sample *t*-test (*n* = 6–8/group). (*n* = 6–8/group)

One-way ANOVA was used to compare the discrimination index (DI) values among experimental groups during training sessions. No significant differences in DI were observed during Training I [(F_4, 34_ = 1.560; *P* = 0.20) or Training II sessions (F_4, 34_ = 0.5581; *P* = 0.69)] in WT (Fig. 3A, B); VAChT KD^HET^ [(F_4, 32_ = 1.330; *P* = 0.28); (F_4, 34_ = 1.594; *P* = 0.19)] (Fig. 3E, F); and VAChT KD^HOM^ mice [(F_4, 28_ = 2.636; *P* = 0.056) (F_4, 26_ = 0.7162; *P* = 0.58)] (Fig. 3I, J).

**Fig. 4:**
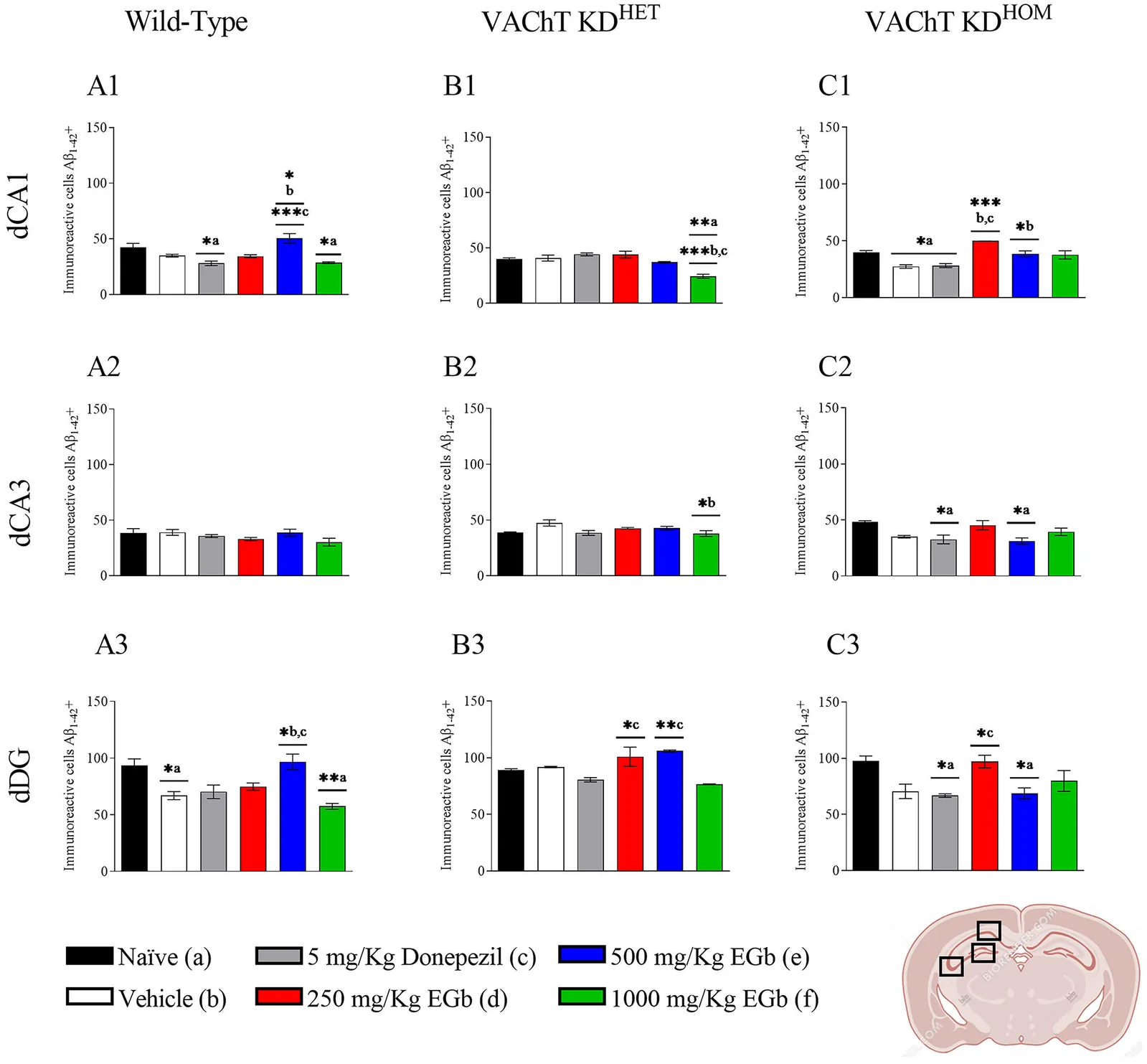
Quantification of monomers of amyloid-β_1−42_ immunoreactive (Aβ-IR^+^) cells in CA1, CA3, and dentate gyrus (DG) of dorsal hippocampal formation of Wild-Type (WT) and both VAChT KD^HET^ and VAChT KD^HOM^ genotypes treated with vehicle, 5 mg/Kg Donepezil or EGb (250, 500 and 1000 mg/Kg). Comparisons between groups are denoted as follows: *P < 0.05, **P < 0.01, and ***P < 0.001 according to One-way ANOVA with post hoc Tukey’s multiple comparison test. Values are expressed as the mean (± SEM) of three fields, each covering an area of 94,566 μm² (*n* = 3–4/group)

During the ORM test, WT mice treated with EGb at a dose of 500 mg/kg (DI = -0.16) exhibited impaired recognition memory, as indicated by significantly lower DIs compared to the vehicle group (DI = 0.15) (F_4, 34_ = 5,222; *P* = 0.0022) (Fig. 3C). One-sample *t*-tests showed that the 500 mg/kg EGb group had a DI significantly below the chance value (t(7) = 3.473; *P* = 0.01), indicating a preference for the familiar object over the novel one. In contrast, mice treated with EGb at 1000 mg/Kg were unable to discriminate the novel object from the familiar object (DI = -0.05); (*t*(7) = 0.9236; *P* = 0.3864). Conversely, the vehicle (DI = 0.16; (*t*(6) = 2.510; *P* = 0.04) and 250 mg/kg (DI = 0.15; *t*(7) = 4.504; *P* = 0.0028) groups showed significantly indices significantly above chance level, indicating successful ORM formation. In the OLM test, one-way ANOVA revealed no significant differences among WT groups (F_4, 34_ = 2.275; *P* = 0.08) (Fig. 3D).

In VAChT KD^HET^ mice, there were no significant differences between groups during ORM test (F_4, 34_ = 0.9381; *P* = 0.45) (Fig. 3G). However, the one-Sample *t*-Test indicated that both the vehicle-treated (*t*(6) = 3.377; *P* = 0.01; DI = 0.22) and 250 mg/kg EGb (*t*(7) = 2.425; *P* = 0.04; DI = 0.20) groups had significantly higher DIs than chance, indicating enhanced interaction with the novel object. In OLM testing, a significant treatment effect was observed (F_4, 34_ = 5.000; *P* = 0.002) with 1000 mg/kg EGb (DI = -0.03) showing impaired location memory compared to the vehicle group (DI = 0.42; *P* = 0.003) (Fig. 3H). One-sample *t*-Test confirmed that the vehicle group had a DI significantly above the chance level (*t*(6) = 4.509; *P* = 0.004; DI = 0.42), indicating successful long-term memory (LTM) formation. The 250 mg/kg EGb group also showed successful LTM formation (*t*(7) = 3.544; *P* = 0.009; DI = 0.31).

No significant differences were found between groups for VAChT KD^HOM^ mice in ORM (F_4, 27_ = 0.8471; *P* = 0.50) (Fig. 3K). However, one-sample *t*-tests showed that EGb 250 mg/kg-treated mice had a DI significantly above chance (*t*(4) = 3.909; *P* = 0.01; DI = 0.18). In the OLM test (Fig. 3L), EGb 250 mg/kg-treated mice exhibited a higher DI (*t*(5) = 2.758; *P* = 0.039; DI = 0.41); compared to the vehicle (*t*(6) = 1.791; *P* = 0.013; DI = -0.18; Cohen’s D = 0.48) and Donepezil (*t*(6) = 0.7993; *P* = 0.041; DI = -0.10) groups, with significant results confirmed by one-way ANOVA (F_4, 28_ = 3.539; *P* = 0.0185). See Supplementary Fig. 1 for more details about the time spent explorations of mice in both ORM and OLM tests.

#### Influence of Aging and Cholinergic Signaling on Anxiety and Spontaneous Locomotor Behavior

We also analyzed the anxiety index of each genotype during the training session. Wild-Type mice (F_4, 33_ = 1.163; *P* = 0.34), VAChT KD^HET^ (F_4, 35_ = 1.447; *P* = 0.23) and VAChT KD^HOM^ (F_4, 25_ = 0.63; *P* = 0.64) displayed similar indices across all groups. Moreover, no significant differences in spontaneous motor activity were observed between groups for Wild-Type (F_2,259, 19,20_ = 02807; *P* = 0.78), VAChT KD^HET^ (F_1,959, 13,71_ = 1.909; *P* = 0.18) and VAChT KD^HOM^ (F_2,786, 17,41_ = 2.355; *P* = 0.11) (see Supplementary Fig. 2 for graphs).

### Molecular Analysis

#### Effects of Aging and EGb on Amyloid-β-42 Expression

Immunohistochemistry analysis revealed a significant effect of treatment in the CA1 subfield of WT mice (Fig. 4A1) (F_5, 12_ =10.58; *P* = 0.0005; Cohen’s d = 0.62). Mice treated with 500 mg/kg EGb showed a higher number of Aβ-immunoreactive cells (Aβ-IR^+^) compared to vehicle and 5 m/kg Donepezil. No significant differences were observed in CA3 subfield (F_5, 12_ = 1.558; *P* = 0.24) (Fig. 4A2). In the DG, there was a significant effect of treatment (F_5, 12_ = 9.805; *P* = 0.0006; Cohen’s d = 0.93) (Fig. 4A3), where mice treated with 500 mg/kg EGb showed increased Aβ-IR^+^ cells compared to those treated with vehicle and Donepezil.

In the CA1 subfield of VAChT KD^HET^ mice (Fig. 4B1), treatment significantly affected Aβ-IR^+^ cells (F_5, 12_ = 13.62; *P* = 0.0001). Mice treated with 1000 mg/kg EGb exhibited reduced Aβ-IR^+^ cells compared to the naïve, vehicle, and Donepezil groups. Similarly, in CA3 (Fig. 4B2), 1000 mg/kg EGb significantly decreased Aβ-IR^+^ cells compared to the vehicle group (F_5, 12_ = 3.595; *P* = 0.03). In the DG (Fig. 4B3), treatment with Donepezil decreased Aβ-IR+ cells compared to 250 and 500 mg/kg EGb groups (F_5, 12_ = 9.810; *P* = 0.0006), but no differences were found between EGb groups and vehicle group.

In VAChT KD^HOM^ mice, both 250 (Cohen’s d = 1.90) and 500 mg/kg (Cohen’s d = 0.87) increased Aβ levels in the CA1 subfield compared to the vehicle group (F_5, 12_ = 14.36; *P* = 0.0001) (Fig. 4C1). Furthermore, the 250 mg/kg EGb group showed significantly higher Aβ levels compared to the Donepezil group. In the CA3 (Fig. 4C2), treatment with 500 mg/kg EGb and Donepezil resulted in lower Aβ levels compared to the naïve group (F_5, 12_ = 5.494; *P* = 0.0074). In the DG (Fig. 4C3), the 250 mg/kg EGb group had higher Aβ levels compared to Donepezil (F_5,12_ = 5.847; *P* = 0.0058). The images obtained from immunohistochemistry can be assessed in Supplementary Fig. 3.

#### Effects of EGb on Amyloid-β Aggregation In Vitro

**Fig. 5:**
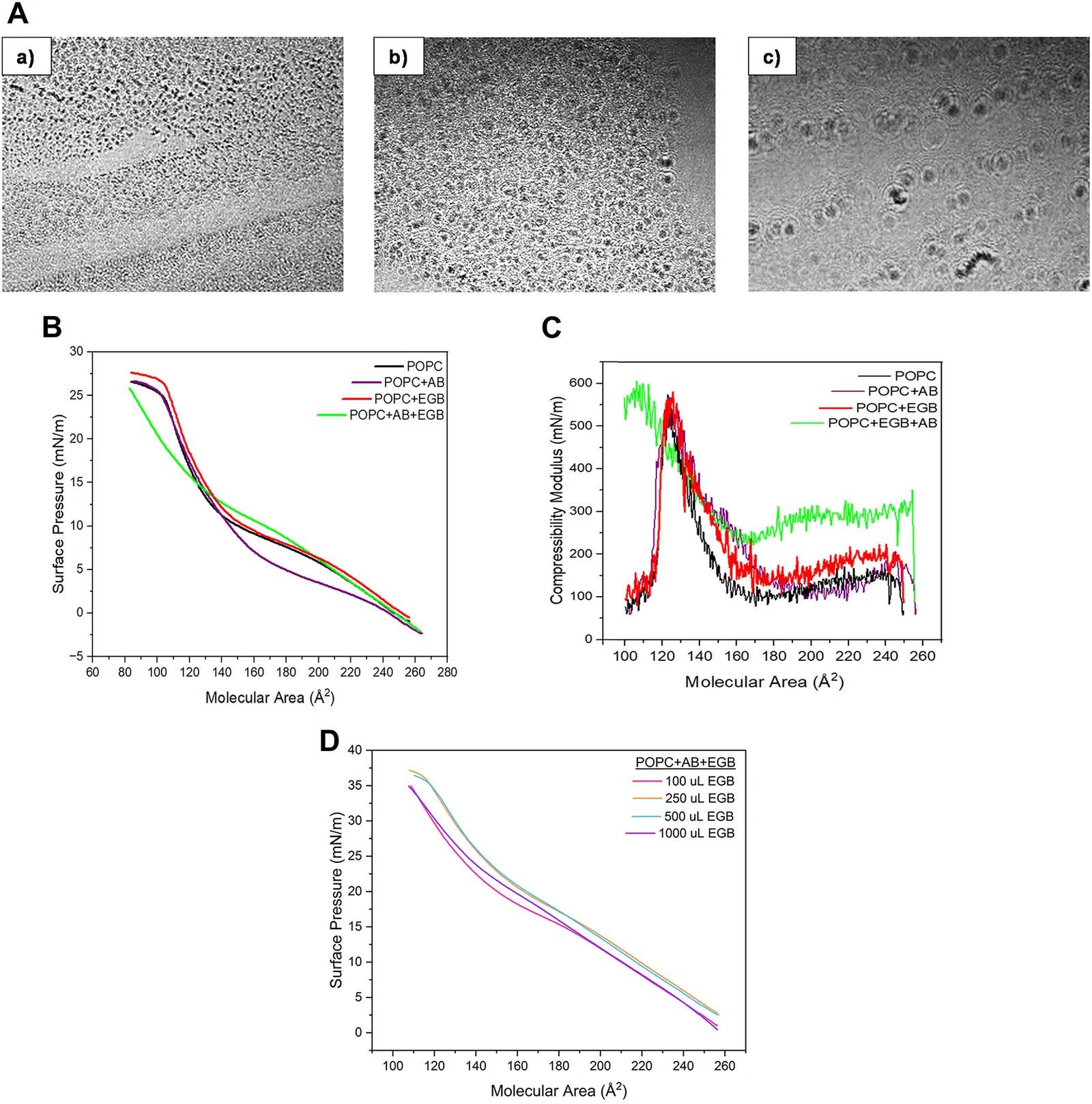
**A** BAM images (3600 × 2400 μm) of POPC monolayers pure (a), with Aβ (b) and with Aβ and EGb (c). BAM image of POPC monolayer at a pressure of 15 mN/m. Surface pressure of 18 ± 2 mN/m. **B** All four curves exhibited similar behavior, regardless of the dosage used. Therefore, it was decided to standardize the experiments using 100 µL of EGb. Isotherms were obtained from pure and Aβ-spread POPC monolayers, with and without EGb in the subphase. **C** Isotherms of pure POPC, POPC with EGb, POPC: Aβ and POPC: Aβ with EGb films. **D** 1st derivative of the isotherms of POPC, POPC with EGb, POPC: Aβ and POPC: Aβ with EGb

Brewster Angle Microscopy (BAM) was employed to visualize the POPC monolayer morphology (Fig. 5), examining the effects of introducing new components on aggregate formation. Initially, POPC on a PBS subphase (Fig. 5Aa) showed small, bright spheres, indicating monolayer formation and pre-collapse aggregates due to rapid compression. Adding Aβ formed larger, darker aggregates (Fig. 5Ab and Ac), increasing density and coalescing while leaving homogeneous domains. Incorporating EGb (Fig. 5Ac) decreased aggregate density, dispersing smaller domains. This suggests EGb’s dispersing action interacts with Aβ, hindering POPC-Aβ cohesion. Surface pressure-area isotherms (Fig. 5B) demonstrate that introducing EGb expands the film in the liquid-expanded phase, indicating monolayer condensation and attractive interactions at higher surface pressures (> 12 mN/m). This implies direct EGb-Aβ interaction. The compressibility modulus (Fig. 5C) indicates that the monolayer containing EGb, Aβ, and POPC exhibits lower surface compressional modulus, indicating fluidization and reduced resistance to compression. Isotherms using varying EGb volumes (100, 250, 500 and 1000 µL) demonstrated expanded liquid regions up to 12 mN/m (Fig. 5D). EGb interacts significantly with Aβ, modifying POPC’s solid region. Analysing the curves confirms EGb’s direct interaction with Aβ. EGb disrupts ordered lipid packing, making the monolayer more fluid and less resistant to compression, highlighting specific EGb-Aβ interactions within a lipid matrix mimicking cell membranes.

#### Effects of EGb on Phosphorylated-Tau Expression

**Fig. 6:**
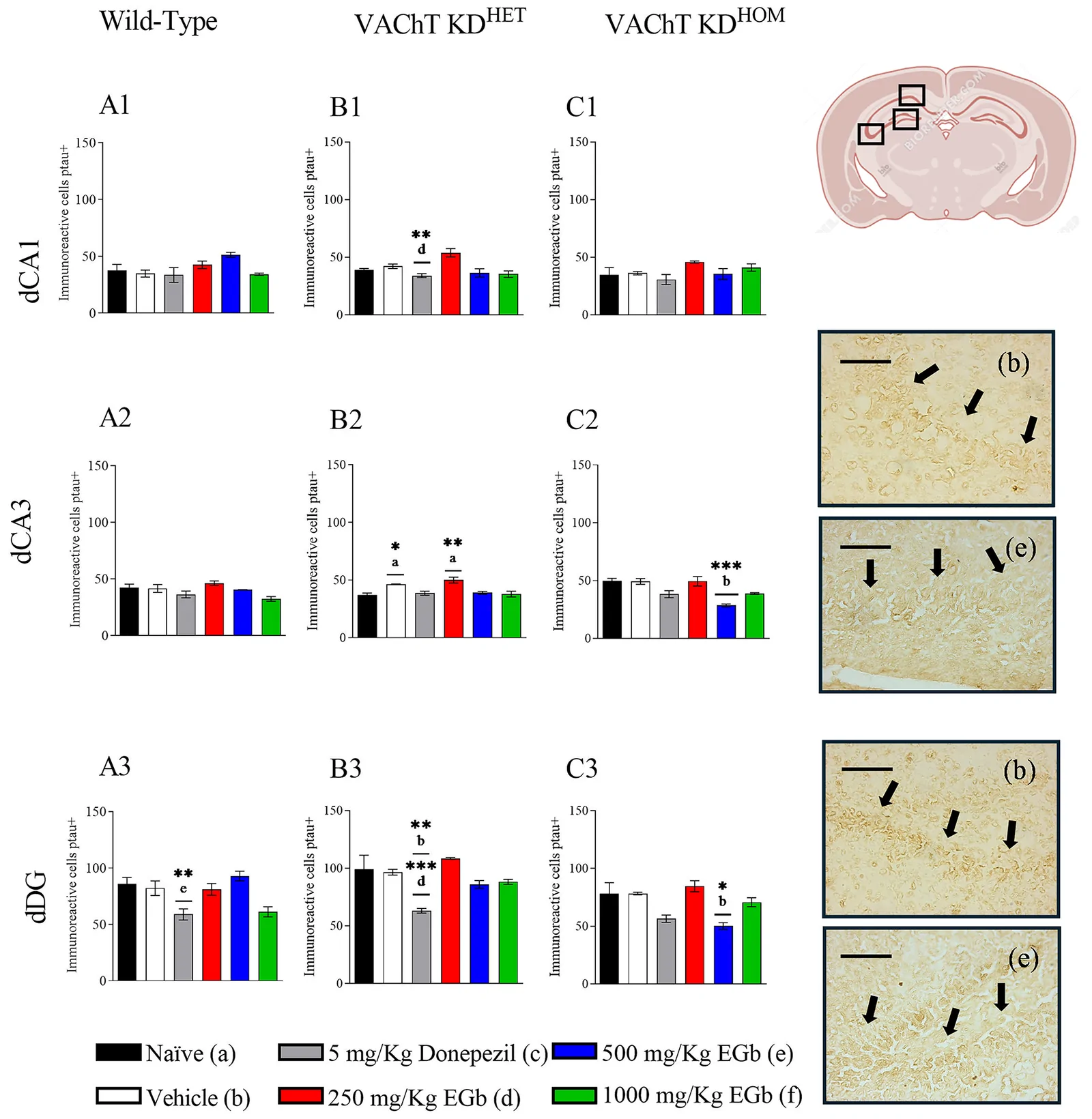
Quantification of pTau immunoreactive cells (pTau-IR^+^ cells) of Wild-Type and both VAChT KD^HET^ and VAChT KD^HOM^ genotypes treated with vehicle, 5 mg/kg Donepezil or EGb (250, 500 and 1000 mg/kg). Comparisons between groups are denoted as follows: *P < 0.05, **P < 0.01, and ***P < 0.001 according to One-way ANOVA with post hoc Tukey’s multiple comparison test. Values are expressed as the mean (± SEM) of three fields, each covering an area of 94,566 μm in the CA1, CA3, and dentate Gyrus (DG) of the dorsal hippocampal formation (HF) (*n* = 3–4/group). In the photomicrographs, the black bar represents 50 μm. Black arrows indicate the cells expressing pTau in the pyramidal cell layer of CA1 and CA3 and the granular layer of DG

No significant effect of treatment was observed in the number of pTau-IR^+^ cells (pTau-IR^+^) in the CA1 subfields of WT animals (F_5, 12_ = 2.904; *P* = 0.06) (Fig. 6A1). However, in the CA3 subfield mice treated with 250 mg/kg EGb exhibited higher pTau-IR^+^ cells compared to those treated with 1000 mg/kg EGb (Fig. 6A2) (F_5, 12_ = 3.578; *P* = 0.032). A similar treatment effect was observed in the DG (F_5, 12_ = 7.023; *P* = 0.0028) (Fig. 6A3), where mice treated with Donepezil group showed decreased pTau-IR^+^ cells compared to the 500 mg/kg EGb group. In VAChT KD^HET^ mice, significant treatment effects were observed in the CA1 (F_5, 12_ = 7.636; *P* = 0.0019) (Fig. 6B1), CA3 (F_5, 12_ = 8.441; *P* = 0.0013) (Fig. 6B2), and the DG (F_5, 12_ = 8.311; *P* = 0.0013) (Fig. 6B3). Specifically, in CA1, mice treated with Donepezil showed decreased number of pTau-IR^+^ cells compared to 250 mg/kg EGb; while in CA3 the EGb 250 mg/ and vehicle group showed higher pTau-IR^+^ cells compared to naïve. Finally, in DG the Donepezil group showed decrease ofpTau-IR^+^ cells when compared to naïve, vehicle and EGb 250 mg/Kg.

Analysis of pTau-IR^+^ cells in VAChT KD^HOM^ mice revealed no treatment effects in the CA1 (F_5, 12_ = 1.849; *P* = 0.1777) (Fig. 6C1). However, in CA3 (F_5, 12_ = 12.06; *P* = 0.0002; Cohen’s d = 1.77) (Fig. 6C2) and DG (F_5, 12_ = 7.366; *P* = 0.0023; Cohen’s d = 1.33) (Fig. 6C3), mice treated with 500 mg/Kg EGb showed fewer pTau-IR^+^ cells compared to both naïve and vehicle groups.

## Discussion

In summary, our results demonstrate for the first time that: (1) Aging negatively affects discriminative avoidance memory, which depends on the integration of spatial and emotional neural representations within the hippocampus; (2) Aging mice associated with hypocholinergic function exhibit similar deficits in aversive memory; (3) Treatment with EGb in a dose-dependent manner effectively reverses impairments in both short- and long-term aversive memory in VAChT KD^HOM^ mice; (4) While aging does not impair ORM in WT animals, it does affect OLM; (5) VAChT KD^HET^ mice can successfully form both ORM and OLM despite aging; (6) VAChT KD^HOM^ exhibit impairments in both ORM and OLM; (7) Lower doses of EGb enhance hippocampal-dependent memories in older female VAChT KD^HOM^ mice, as evaluated through discriminative avoidance task and both ORM and OLM; 7) Lower doses of EGb increase the number of Aβ monomer-positive decreasing pTau IR^+^ cells in the dCA3 and dDG regions of VAChT KD^HOM^ mice. 9) Our in vitro analysis shows that EGb interacts with Aβ and POPC monolayers, shedding light on its potential role in the formation of Aβ plaques in AD.

The deficits in spatial memory formation, specifically in discriminative avoidance memory and OLM, observed in older mice are supported by literature indicating that normative aging is associated with physiological and anatomical changes in HF [7]. In the PMDAT task, WT mice were unable to remember the aversive enclosed arm. Our findings are consistent with those of Leão and collaborators [58], who demonstrated that both proximal and distal cues are required for the formation of aversive memory in PMDAT, with proximal cues playing a predominant role in spatial orientation. Together with previous findings demonstrating that transient inactivation of the dorsal CA1 (dCA1) impairs the retrieval of long-term discriminative avoidance memory [59], the present results suggest that the age-related impairment observed in WT mice may result, at least in part, from functional alterations within the dCA1. Thus, our findings extend previous observations by providing evidence that aging compromises a hippocampal circuit already shown to be essential for the acquisition and retrieval of aversive spatial memory in the PMDAT. Neurons in the cortex and hippocampus are especially sensitive to the aging process [2, 7]. Although the CA1 subfield shows relative resistance to aging-related changes, we previously demonstrated that deficits in aversive long-term memory formation are associated with reduced recruitment of neuronal ensembles within the dorsal CA1 (dCA1) subfield [14]. Building on these findings, the present results suggest that age-related impairments in PMDAT performance may arise, at least in part, from functional alterations within this hippocampal circuit. In addition, our cellular analyses suggest that the dentate gyrus (DG) may also contribute to these deficits in conjunction with the CA1 region. This interpretation is supported by evidence that the DG is one of the earliest hippocampal subregions affected during aging, where reduced Arc protein expression has been associated with age-related impairments in memory consolidation [60].

Regarding OLM, aged WT mice failed to recognize the object in a novel location, reinforcing the well-established impact of aging on hippocampal-dependent spatial memory. In contrast, ORM remained preserved, suggesting that aging preferentially affected hippocampal function while sparing perirhinal cortex-dependent recognition memory [61]. This dissociation also argues against a major influence of the behavioral testing sequence, as a nonspecific carryover effect of the preceding Plus-Maze Discriminative Avoidance Task (PMDAT), including stress- or novelty-related responses such as neophobia, would be expected to affect performance in both recognition memory tasks. Importantly, the PMDAT induces only moderate acute emotional arousal rather than intense or prolonged stress, as its aversive component is restricted to exposure to light and noise in one enclosed arm of the maze [62]. Furthermore, ORM and OLM assessments were performed 72 h after PMDAT, a time interval well beyond the period during which acute stress is known to influence memory processing. Consistent with this interpretation, previous studies have demonstrated that the effects of acute stress on recognition memory are critically dependent on its temporal proximity to memory acquisition, consolidation, or retrieval, with little or no impact observed when stress exposure precedes behavioral testing by several hours or days [63–66]. Together, these findings indicate that prior PMDAT exposure was unlikely to have significantly influenced subsequent ORM or OLM performance. Nevertheless, behavioral testing order should be considered when interpreting the present findings.

In contrast, VAChT KD^HET^ mice showed preserved performance in both ORM and OLM, suggesting that a mild chronic reduction in VAChT levels may engage compensatory mechanisms within cholinergic and associated hippocampal and extrahippocampal circuits. Similar adaptative responses have been described during the early stages [67, 68]. In WT mice, however, such adaptive mechanisms may emerge later in life and appear to be insufficient to prevent the age-related decline in hippocampal-dependent spatial memory observed in the present study.

Regarding VAChT KD^HOM^, our findings align with previous studies highlighting the crucial role of cholinergic neurotransmission in hippocampal-dependent memory [43, 69, 70]. These animals showed deficits in both ORM and OLM, indicating a broad impairment in recognition memory. Notably, these deficits were independent of aging, as young and older animals exhibited similar performance across tasks, supporting a genotype-driven effect. Although a reduced or negative discrimination index (DI) is often interpreted as evidence of impaired recognition memory, motivational and/or exploratory factors, including neophobia, may also contribute to the absence of novelty preference. Therefore, the interpretation of recognition memory performance was based not only on discrimination index values, but also on statistical comparisons against chance level (DI = 0) and on the overall behavioral profile across tasks and experimental groups.

Our data demonstrated that EGb enhances both STM and LTM in a dose-dependent manner in VAChT KD^HOM^ mice, as assessed by the PMDAT during training and test sessions. The beneficial effect on LTM was further supported by latency to the first entry into the aversive enclosed arm (AEA) during the test session. Similarly, EGb at a dose of 250 mg/kg reversed the impairments observed in ORM and OLM in VAChT KD^HOM^ mice. These findings are consistent with our previous study, in which the same dose of EGb effectively reversed deficits in OLM in young female VAChT KD^HOM^ mice [42], supporting the reproducibility of its beneficial effects on hippocampal-dependent memory.

The cognitive-enhancing effects of EGb have been previously demonstrated by our research group [39–41, 48]. Additionally, EGb not only enhances STM but also prevents oxidative damage in the prefrontal cortex and hippocampus of middle-aged rats (12 months) subjected to PMDAT [37]. Another potential mechanism involved in this process, beyond our cellular analysis findings, is brain-derived neurotrophic factor (BDNF). Our research indicates that EGb modulates BDNF levels, which play a crucial role in neuroprotection and the maintenance of long-term memory (LTM). Conversely, EGb at doses of 500 and 1000 mg/kg failed to reverse deficits in long-term memory in older wild-type (WT) mice and older mice with severe hypocholinergic dysfunction (VAChT KD^HOM^), as assessed by the PMDAT, ORM, and OLM tasks.

These effects may be linked to EGb effects on Aβ-IR^+^ cells and pTau levels in the dHF. Since EGb at a dose of 250 mg/kg increased Aβ monomer expression and reversed memory impairments, this suggests that EGb may modulate Aβ dynamics by preventing its aggregation into larger protein assemblies, such as oligomers. Our in vitro analysis supports this suggestion. Data from Fig. 5 demonstrate that EGb positively affects the interaction with the Aβ/POPC monolayer. Typically, Aβ tends to form aggregates when adsorbed onto the lipid monolayer; however, the introduction of EGb into the subphase causes the molecules to disperse, resulting in a more uniform distribution at the interface. These in vitro results directly reinforce our in vivo findings. In this context, the interaction of EGb with the Aβ and POPC monolayer may help clarify its role in the formation of Aβ plaques in Alzheimer’s disease. Furthermore, studies have shown that EGb exerts effects against Aβ by interfering with oligomerization and fibril formation [71, 72].

Recent evidence suggests that amyloid-β and tau pathologies are not independent processes; rather, they engage in synergistic and mutually reinforcing interactions. Aβ-induced synaptic hyperexcitability may promote tau mislocalization, phosphorylation, and spread, while tau can further exacerbate Aβ-mediated neuronal dysfunction, establishing a feed-forward loop that accelerates synaptic failure and cognitive decline [73]. Within this complex interplay, tau represents a critical downstream effector of Aβ-driven network dysfunction. Accordingly, modulation of Aβ may indirectly influence tau pathology through changes in neuronal activity and intracellular signaling pathways, rather than acting exclusively at the level of tau expression. In this framework, the cognitive effects observed following EGb treatment may reflect not only direct modulation of Aβ and phosphorylated tau levels, but also broader effects on neuronal network activity and intracellular signaling cascades that regulate Aβ–tau interactions. Therefore, the relationship between behavioral performance and individual neuropathological markers is likely multifactorial rather than linear. Future studies specifically designed to correlate cognitive outcomes with detailed histopathological and functional measures will be important to further clarify these mechanistic relationships.

Previous studies have highlighted the neuroprotective properties of EGb, including its reported effects on tau pathology, although these effects appear to vary depending on the experimental model and phosphorylated epitope analyzed [74].

Tau plays a crucial role in brain physiology and may exert distinct effects depending on its isoforms [75]. It is predominantly located in neuronal axons, where it regulates microtubule stability and axonal transport [76], and its phosphorylation state is tightly controlled by the balance between kinase and phosphatase activities. Disruption of this balance leads to hyperphosphorylation, detachment from microtubules, cytoskeletal instability and subsequent aggregation into oligomers and neurofibrillary tangles [74]. In this context, flavonoid compounds have been shown to interfere with tau aggregation processes, including disruption of preformed aggregates and paired helical filaments, which are precursors of neurofibrillary tangles [77].

Previous studies have also highlighted EGb’s neuroprotective properties, including its modulation of Tau, which appears to depend on the specific phosphorylated tau epitope [78]. Phosphorylation at threonine 231 (pTau2T31) has been associated with early AD-related pathological changes and is detectable in both cerebrospinal fluid and plasma, supporting its potential use as an early biomarker. A recent review further indicates that Thr231 is one of the key phosphorylation sites involved in tau aggregation, neurofibrillary tangle formation, and synaptic dysfunction. p-TauT231 is particularly valuable for identifying early amyloid-related changes before significant plaque formation [79].

Interestingly, in our study, we observed increased p-Tau Thr231 expression in VAChT KD^HET^ mice in the dentate gyrus compared to VAChT KD^HOM^ animals (data not shown), suggesting a possible association between cholinergic status and early tau-related alterations. This observation is consistent with evidence indicating that p-Tau Thr231 is more prominent in early or prodromal stages of AD [80]. However, EGb-specific effects on this phosphorylation site were not directly assessed and remain to be determined.

Although the cholinergic impairment of VAChT KD mice has been extensively characterized in previous studies, the present study did not directly quantify acetylcholine levels or other cholinergic neurochemical markers. Instead, we focused on determining whether EGb could attenuate Alzheimer’s disease-related pathological markers, demonstrating reductions in Aβ and phosphorylated Tau immunoreactivity in association with improved cognitive performance. These findings suggest that the observed behavioral effects may be linked not only to amyloid and tau modulation, but also to broader neurobiological mechanisms that were not directly assessed in the present work. Future studies should therefore investigate whether these neuroprotective effects are accompanied by restoration of cholinergic neurotransmission, including assessment of acetylcholine content, cholinergic enzyme activity, and cholinergic receptor expression in VAChT KD mice. Such approaches will help clarify whether modulation of the cholinergic system contributes mechanistically to the beneficial effects of EGb on AD-related neuropathology.

In this context, a previous study has demonstrated that *G. biloba* extract exerts neuroprotective and anti-apoptotic effects against scopolamine-induced neurotoxicity [81]. The present findings extend these observations by demonstrating beneficial effects in a genetic model of chronic cholinergic hypofunction, suggesting that the therapeutic potential of *G. biloba* is not restricted to acute muscarinic receptor blockade.

## Conclusions and future directions

Our comprehensive analysis demonstrates that the 250 mg/kg dose of EGb reverses cognitive deficits in KD^HOM^ mice, as evidenced by improvements in both behavioral tasks. The increase in Aβ expression in neural cells of the pyramidal layer of dCA1 suggests that this dose may either disrupt existing aggregates or prevent their formation altogether. Furthermore, our findings underscore the pivotal role of modulating both pTau and Aβ monomers in mitigating the effects of aging and cholinergic dysfunction in older female mice. AD is characterized by the accumulation of Aβ plaques in the extracellular space and the formation of tau-containing neurofibrillary tangles, which together contribute to neuronal dysfunction and glial cell activation. In this context, our results suggest that those pathological processes are closely linked to aging and cholinergic deficits in female mice. Furthermore, for the first time, we propose a potential mechanism through which EGb may modulate these pathways. These results emphasize the importance of exploring the alterations in neuronal and glial pathways, their interactions, and the potential involvement of estrogen in these mechanisms.

## Supplementary Information

Below is the link to the electronic supplementary material.

Supplementary Material 1

Supplementary Material 2

Supplementary Material 3

Supplementary Material 4

## Data Availability

All data supporting the findings of this study are available within the paper and its Supplementary Information.

## Abbreviations

AD: Alzheimer’s disease
Ach: Acetylcholine
VAChT: Vesicular Acetylcholine Transporter
(LOAD: Late-onset Alzheimer’s Disease
HF: Hippocampal Formation
DHF: Dorsal Hippocampal Formation
EC: Entorhinal Cortex
EGb: Standardized Extract of *Ginkgo biloba*
KD: Knockdown
HET: Heterozygous
HOM: homozygous
WT: Wild type
PMDAT: Plus-maze discriminative avoidance task
OLM: Object location memory
ORM: Object recognition memory
LTM: Long-term memory
STM: Short-term memory
CA1: Cornu Ammonis 1
CA3: Cornu Ammonis 3
DG: Dentate Gyrus
dCA1: Dorsal Cornu Ammonis 1
dCA3: Dorsal Cornu Ammonis 3
dDG: Dorsal Dentate Gyrus
DPZ: Donepezil
Aβ: Amyloid-β
% tAEA: Percentage of time spent in the aversive enclosed arm
% tOA: Percentage of time spent in the open arm
% eOA: Percentage of entries in the open arm
pTau: Phosphorylated tau
Ca^2+^: Calcium ion
PBS: Phosphate buffer solution
POPC: 1-Palmitoyl-2-oleoyl-sn-glycerol-3-phosphocholine
M1mAChR: M1muscarinic cholinergic receptors
ERα: Strogen receptor alpha
