# Supplementary figures and images for: Standardized Extract of Ginkgo biloba L. Reverses Memory Impairment in Older Female Mice with Basal Forebrain Cholinergic Dysfunction
Source: Neurochem Res. 2026 Jul 14;51(4):217. doi: 10.1007/s11064-026-04829-0 (PMC13364894; doi:10.1007/s11064-026-04829-0)

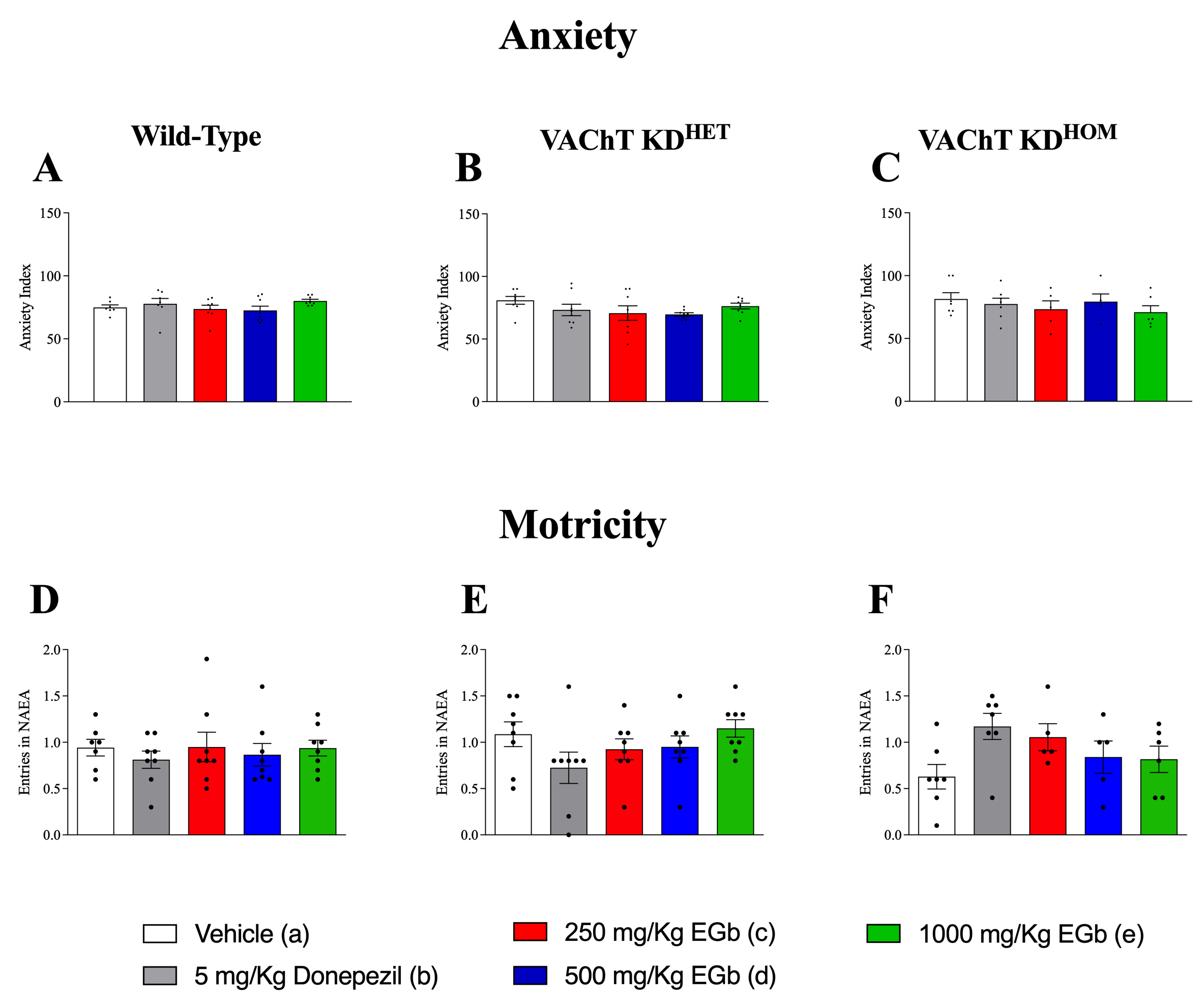

Supplement: Supplementary file 2 — Supplementary Material 2 [file 11064_2026_4829_MOESM2_ESM.tiff]

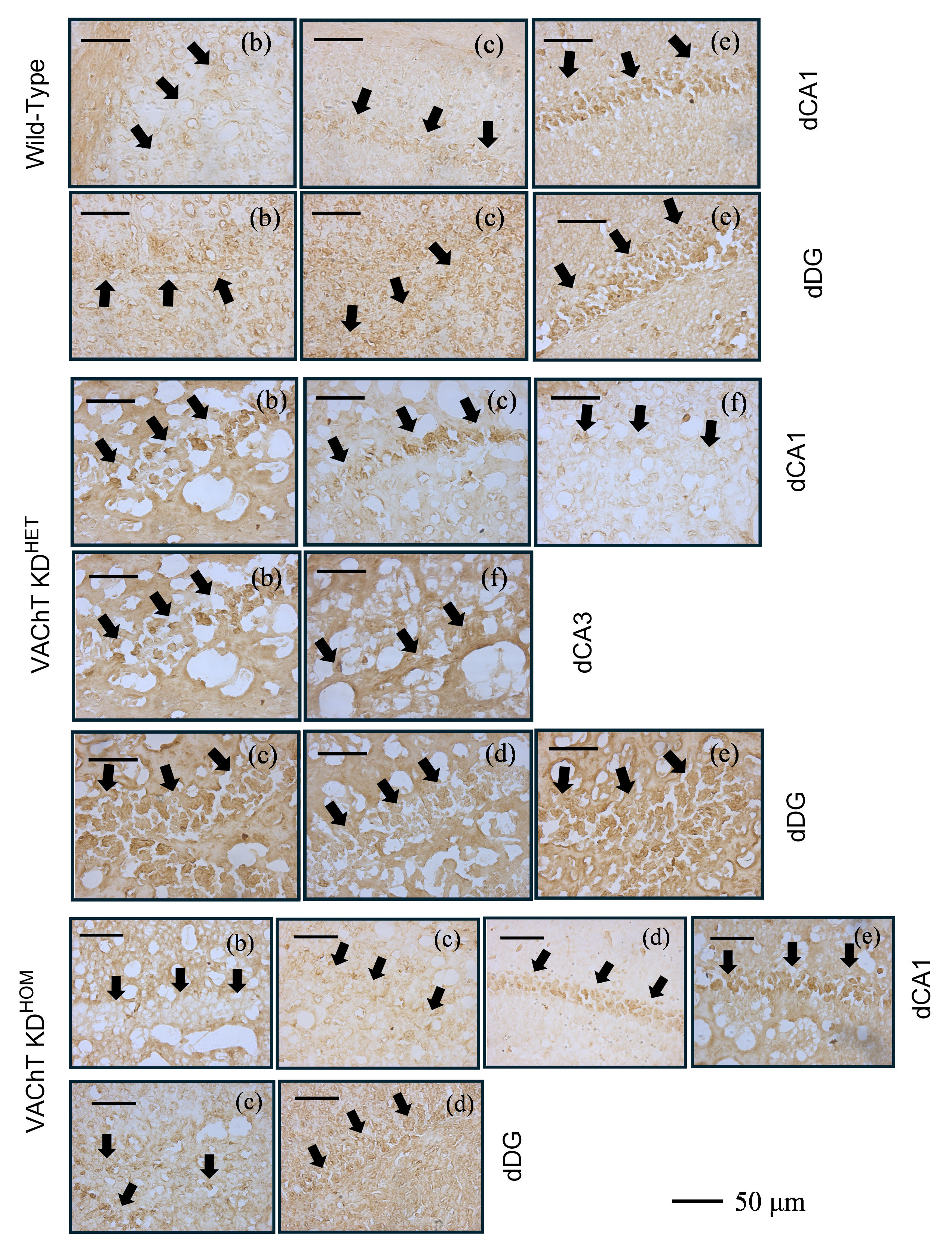

Supplement: Supplementary file 3 — Supplementary Material 3 [file 11064_2026_4829_MOESM3_ESM.tiff]
