# Supplementary material for: Standardized Extract of Ginkgo biloba L. Reverses Memory Impairment in Older Female Mice with Basal Forebrain Cholinergic Dysfunction
Source: Neurochem Res. 2026 Jul 14;51(4):217. doi: 10.1007/s11064-026-04829-0 (PMC13364894; doi:10.1007/s11064-026-04829-0)
Supplement: Supplementary file 4 — Supplementary Material 4 [file 11064_2026_4829_MOESM4_ESM.docx]

**Supplementary Material**

**3. Results**

*3.2 EGb enhances hippocampal-dependent memory in older mice in a dose-dependent manner*

*3.2.2 Non-aversive memory*

In training sessions, WT animals and both KD mice explored the sample objects equally according to a paired *t-*test. For WT mice in training session I: vehicle t(5) = 1.442; *P*= 0.208; Donepezil t(5) = 0.006272; *P*= 0.995; EGb 250 t(5) = 0.6489; *P*= 0.545; EGb 500 t(6) = 0.8188; *P*= 0.444 and EGb 1000 t(7) = 0.7232; *P*= 0.493. In training session II: vehicle t(4) = 0.9257; *P*= 0.407; Donepezil t(6) = 1.366; *P*= 0.221; EGb 250 t(6) = 1.263; *P*= 0.253; 500 t(7) = 1.134; *P*= 0.294 and EGb 1000 t(7) = 0.3680; *P*= 0.723 (Supplementary figure 2A-B).

For VAChT KD^HET^ in training session I: vehicle t(5) = 1.419, *P*= 0.2151; Donepezil t(8) = 0.2101; *P*= 0,838; EGb 250 t(7) = 0.2252; *P*= 0.828; EGb 500 t(7) = 0.06978; *P*= 0.946 and EGb 1000 t(7) = 0.7344; *P*= 0.486. In training session II: vehicle t(6) = 2.246; *P*= 0.065; Donepezil t(7) = 1.081; *P*= 0.3155; EGb 250 t(7) = 0.5094; *P*= 0.626; EGb 500 t(5) = 1.325; *P*= 0.242 and EGb 1000 t(7) = 0.04261; *P*= 0.967 Supplementary figure 2E-F).

Finally, for VAChT KD^HOM^ mice in training session I: vehicle t(5) = 1.887;*P*= 0,1177; Donepezil t(6) = 0.3399; *P*= 0.74; EGb 250 t(4) = 1.865; *P*= 0.135; EGb 500 t(5) = 2.548; *P*= 0.0514 and EGb 1000 t(5) = 1.642; *P*= 0.161 and training session II: vehicle t(4) = 0.5779; *P*= 0.594; Donepezil t(5) = 0.1685; *P*= 0.872; EGb 250 t(5) = 0.2019; *P*= 0.253; EGb 500 t(6) = 0.9414; *P*= 0.3828 and EGb 1000 t(5) = 1.520; *P*= 0.189 (Supplementary figure 2I-J).

In test sessions, a paired-*t* test showed that VAChT KD^HET^ explored the new object more when compared to the new one according to the paired-*t* test when treated with vehicle [t(6) = 2.760; *P* = 0.032] and EGb 250 [t(7) = 2.453; *P* = 0.043] in ORM. The same occurred in OLM for mice treated with vehicle [t(6) = 3.171; *P* = 0.012] and EGb 250 [t(7) = 2.935; *P* = 0.021] (Supplementary figure 2G-H). No statistical differences were found for WT (Supplementary Figure 2C-D) and KD^HOM^ (Supplementary Figure 2K-L) mice in that analysis according to a paired *t*-test.

**Supplementary Figures**

**Supplementary Figure 1.** Time spent exploring the objects in both training sessions (I and II) and in both test sessions (ORM and OLM) for WT, VAChT KD^HET^ and VAChT KD^HOM^ treated with vehicle, 5 mg/Kg donepezil and EGb at doses: 250, 500 and 1000 mg/Kg. Significant differences are indicated (*P < 0.05) based on a paired *t-*test (n = 6-8).

**Supplementary Figure 2.** Anxiety index and percentage of time spent in the non- aversive enclosed arm (NAEA) during the training and test sessions of PM-DAT for WT (A and D), VAChT KD^HET^ (D and E), and VAChT KD^HOM^ (C and F) mice treated with vehicle, EGb (250, 500, or 100 mg/kg), or 5 mg/kg Donepezil. Significant differences between groups are indicated (*P < 0.05) based on two-way ANOVA with multiple comparisons (n = 6–8). The anxiety index was calculated as the mean of the proportion of time spent in the open arms (time in open arms/total time) and the proportion of entries into the open arms (open arm entries/total entries), multiplied by 100.

**Supplementary Figure 3.** Representative photomicrographs of monomers of amyloid-β_1-42_ (Aβ)-IR cells within the subfields of the dCA1 and dDG of WT, KD^HET^, and KD^HOM^ naïve mice (a) or mice treated with vehicle (0.9% saline) (b), 5 mg/kg Donepezil (c), or EGb at doses of 250 mg/kg (d), 500 mg/kg (e), and 1000 mg/kg (f). The black bar represents 50 μm. Black arrows indicate the cells expressing monomers of Aβ_1-42_ in the pyramidal cell layer of CA1 and CA3 and granular layer of DG.
